# Linking Sfl1 Regulation of Hyphal Development to Stress Response Kinases in Candida albicans

**DOI:** 10.1128/mSphere.00672-19

**Published:** 2020-01-15

**Authors:** Ohimai Unoje, Mengli Yang, Yang Lu, Chang Su, Haoping Liu

**Affiliations:** a Department of Biological Chemistry, School of Medicine, University of California, Irvine, Irvine, California, USA; University of Texas Health Science Center

**Keywords:** *Candida albicans*, hyphal formation, Sfl1

## Abstract

Candida albicans is an important human pathogen responsible for causing both superficial and systemic infections. Its ability to switch from the yeast form to the hyphal growth form is required for its pathogenicity. Acidic pH inhibits hyphal initiation, but the nature of the mechanism for this inhibition is not completely clear. We show that acidic pH represses hyphal initiation independently of the temperature- and farnesol-mediated Nrg1 downregulation. Using a collection of transcription factor deletion mutants, we observed that the *sfl1* mutant induced hyphae in acidic pH but not in farnesol at 37°C. Furthermore, transcription of hyphal regulators *BRG1* and *UME6* was not induced in wild-type (WT) cells but was induced in the *sfl1* mutant during hyphal induction in acidic pH. Using the same screening conditions with the collection of kinase mutants, we found that deletions of the core stress response mitogen-activated protein (MAP) kinase *HOG1* and its kinase *PBS2,* the cell wall stress MAP kinase *MKC1*, and the calcium/calmodulin-dependent kinase *CMK1* allowed hyphal initiation in acidic pH. Furthermore, Hog1 phosphorylation induced by high osmotic stress also retarded hyphal initiation, and the effect was abolished in the *sfl1* and three kinase mutants but was enhanced in the phosphatase mutant *ptp2 ptp3*. We also found functional associations among Cmk1, Hog1, and Sfl1 for cation stress. Our study results suggest that robust hyphal initiation requires downregulation of both Nrg1 and Sfl1 transcriptional repressors as well as timely *BRG1* expression. Acidic pH and cationic stress retard hyphal initiation via the stress-responsive kinases and Sfl1.

**IMPORTANCE** Candida albicans is a commensal as well as a pathogen of humans. C. albicans is able to mount a cellular response to a diverse range of external stimuli in the host and switch reversibly between the yeast and hyphal growth forms. Hyphal development is a key virulence determinant. Here, we studied how C. albicans senses different environmental signals to control its growth forms. Our study results suggest that robust hyphal development requires downregulation of two transcriptional repressors, Nrg1 and Sfl1. Acidic pH or cationic stress inhibits hyphal formation via stress-responsive kinases and Sfl1.

## Abstract (precis)

Candida albicans is a commensal as well as a pathogen of humans. C. albicans is able to mount a cellular response to a diverse range of external stimuli in the host and switch reversibly between the yeast and hyphal growth forms. Hyphal development is a key virulence determinant. Here, we studied how C. albicans senses different environmental signals to control its growth forms. Our study results suggest that robust hyphal development requires downregulation of two transcriptional repressors, Nrg1 and Sfl1. Acidic pH or cationic stress inhibits hyphal formation via stress-responsive kinases and Sfl1.

## INTRODUCTION

Candida albicans is a commensal fungus that is part of the normal human microbiota. It can also cause infection when hosts have a compromised immune system, microbial imbalance, or damaged epithelial lining (1). *Candida* infections can be superficial on the skin and mucosal surfaces or systemic when the fungus disseminates through the bloodstream and colonizes vital organs. While superficial infections are relatively harmless, systemic infections can be life-threatening, with mortality rates of up to 40% (2). The prevalence of C. albicans infections has resulted in an increased interest in understanding how this fungus can adapt to different host niches and colonize and infect different mucosal surfaces and organs.

Within the human host, C. albicans cells are exposed to a variety of conditions, including various pH levels. C. albicans can colonize the stomach (pH 2) (3), vagina (pH 4 to pH 5.5) (4), mouth (pH 6 to 7) (5), and intestines of the gastrointestinal (GI) tract (pH 8). The pH of blood and tissues is slightly alkaline (pH 7.4). Environmental pH influences many key C. albicans biological functions and processes, such as filamentation (6, 7), nutrient acquisition (8, 9), white-opaque switching (10), and cell wall remodeling (11). The conserved Rim101 pathway is the major pH response pathway. Neutral-alkaline pH is sensed by receptors on the plasma membrane, leading to activation of the pH-responsive transcription factor Rim101 via a proteolytic cleavage at its C terminus (12). Mutants of the Rim101 signaling pathway show a growth defect in alkaline pH (12, 13), a defect in filamentation, and reduced virulence (14). Activation of Rim101 promotes the expression of genes for acquisition of nutrients, such as iron (14), and of cell wall genes that facilitate survival within the host (15). In addition to the Rim101 pathway, the calcineurin-dependent Crz1 pathway acts in parallel for adapting to growth in alkaline pH (7). How acidic pH inhibits filamentation is not clear. Genetic data have pointed to the possibility of repression by both Rim101 and Crz2, which acts independently of calcineurin (7).

The ability of C. albicans to switch between a unicellular yeast form and a filamentous form is essential to its survival within its human host (16, 17). Hyphal development has been shown to facilitate escape from the macrophages (18) and is strongly influenced by signals and growth conditions common in the host, such as temperature (19), serum (20), pH (6), hypoxia and 5% CO_2_ (21–25), and *N*-acetylglucosamine (26, 27). Induction of hyphal development has two phases: initiation and maintenance (28, 29). The initiation step involves transient downregulation of the transcriptional repressor Nrg1 mediated by a by temperature shift to 37°C and inoculation of overnight cells to a fresh culture. Elevated temperature (37°C) promotes the transcriptional downregulation of *NRG1*, and this regulation requires the cAMP/protein kinase A (cAMP/PKA) pathway (28, 30). Inoculation dilutes out the quorum sensing molecule farnesol, leading to Nrg1 degradation (30). Endogenous nitric oxide is also important for hyphal initiation via Nrg1 degradation (31). Hyphal maintenance is dependent on the growth medium and environmental conditions. Growth under nutrient-poor conditions or in the presence of serum promotes the expression and binding of transcription factor Brg1 to the promoters of hyphal genes, leading to chromatin remodeling and gene expression (28, 29, 32, 33). Hypoxic conditions combined with 5% CO_2_ maintain hyphae by stabilizing the hypha-specific Ume6 transcription factor important for hyphal maintenance (23, 24, 34, 35). While Nrg1 is the most extensively studied repressor of hyphal development, several other factors have been also shown to inhibit hyphal initiation, including Sfl1. The *sfl1* mutant shows an increased trend of hyphal formation under many conditions (36, 37) and has been shown to target several hyphal transcription factors to repress hyphal formation (36, 38). However, what regulates Sfl1 is not known. In addition to growth-promoting and nutrient-sensing pathways, mitogen-activated protein (MAP) kinases that response to cell wall and/or cell membrane stresses are also involved in hyphal development. These include the high-osmolarity glycerol (HOG) pathway, which allows adaptation to high-osmolarity conditions, oxidative and heavy metal stresses (39–41), and cell wall stresses (42–44). The nature of their relationship with Nrg1 or Sfl1 in hyphal initiation is not clear.

In this study, we revealed that acidic pH inhibits hyphal initiation via a mechanism that is independent of Nrg1 downregulation. By screening the transcription factor and kinase deletion collections for mutants that can filament in acidic pH, we found that deletions of *SFL1*, the core stress response MAP kinase *HOG1* and its kinase *PBS2*, the cell wall integrity MAP kinase *MKC1*, and the calcium/calmodulin-dependent kinase *CMK1* all resulted in hyphal initiation in acidic pH. The relationships of Sfl1 to these kinases and Nrg1 are discussed here.

## RESULTS

### Acidic pH does not block Nrg1 downregulation when cells are inoculated into fresh medium at 37°C.

**FIG 1:**
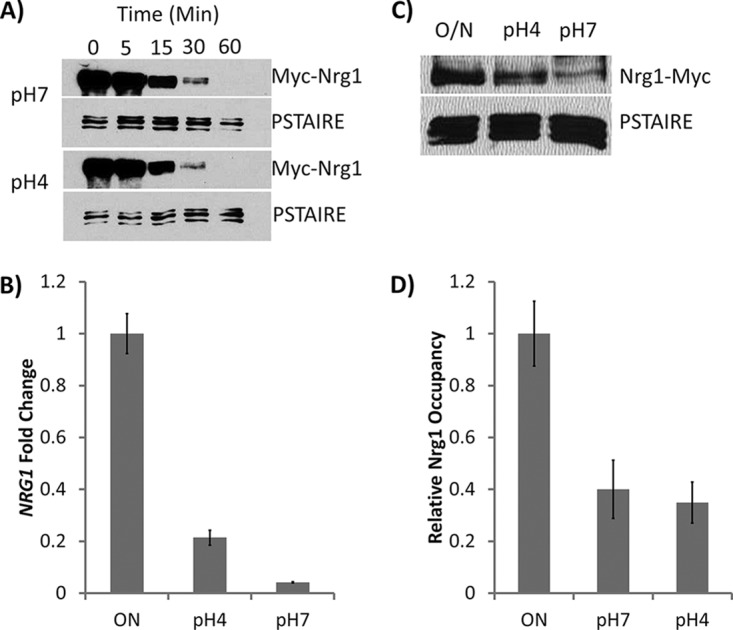
(A) Promoter shutdown assay to compare the levels of Nrg1 stability at pH 4 and pH 7 with those of the WT strain containing a copy of *MAL2p-NRG1-MYC*. A parallel blot was probed with anti-PSTAIRE antibody as a loading control. (B) Reverse transcription-quantitative PCR (qRT-PCR) of *NRG1* transcript level after WT cells were grown at pH 4 and pH 7 for 1 h. Quantitative PCR (qPCR) values were normalized to *ACT1* values for each sample, and overnight (ON) samples were set to a value of 1. Presented data represent means ± standard errors of the means (SEM) of results from 3 independent experiments. (C) Western blot analysis of Nrg1-myc protein levels after WT cells were inoculated into fresh medium at pH 4 and pH 7 for 1 h. ON, overnight. (D) Chromatin immunoprecipitation (ChIP) analysis of Nrg1 for the promoter of *HWP1* after 30 min in YPD medium at pH 4 and pH 7. Presented data represent means ± SEM of results from 3 independent experiments.

Acidic pH is known to inhibit hyphal formation (45), but the mechanism for this inhibition is not completely understood. Hyphal initiation requires rapid removal of the transcriptional repressor Nrg1 by both transcriptional downregulation and protein degradation during hyphal induction, leading to dissociation of Nrg1 from the promoters of hyphal genes (28, 30). To determine if acidic pH inhibits hyphal initiation via blocking the removal of Nrg1 inhibition of hyphal initiation, we examined Nrg1 stability, levels of *NRG1* transcription, and promoter association during hyphal initiation. Cells from overnight culture were inoculated into fresh medium at 37°C and pH 4 or pH 7. Nrg1 protein was similarly degraded under conditions of acidic pH and neutral pH (Fig. 1A), suggesting that acidic pH does not interfere with Nrg1 degradation. *NRG1* transcription was also downregulated at both acidic pH and neutral pH, although the level of its downregulation was not as complete at acidic pH as at neutral pH (Fig. 1B). Consistent with the results shown in Fig. 1A and B, Nrg1 protein levels decreased during hyphal initiation under both pH conditions, but the level at acidic pH was higher than at neutral pH (Fig. 1C). Despite some differences in Nrg1 protein levels, Nrg1 dissociated from the promoter of hyphal gene *HWP1* equally at pH 4 and pH 7 when cells were inoculated into fresh medium at 37°C (Fig. 1D). This suggests that Nrg1-mediated repression of hyphal transcription is not the major regulatory mechanism used by acidic pH. Acidic pH likely acts through a pathway that is different from the pathway of farnesol-mediated Nrg1 degradation or from that of temperature-induced and cAMP/PKA-dependent transcriptional downregulation of *NRG1*.

### The *sfl1* mutant undergoes hyphal initiation in acidic pH.

**FIG 2:**
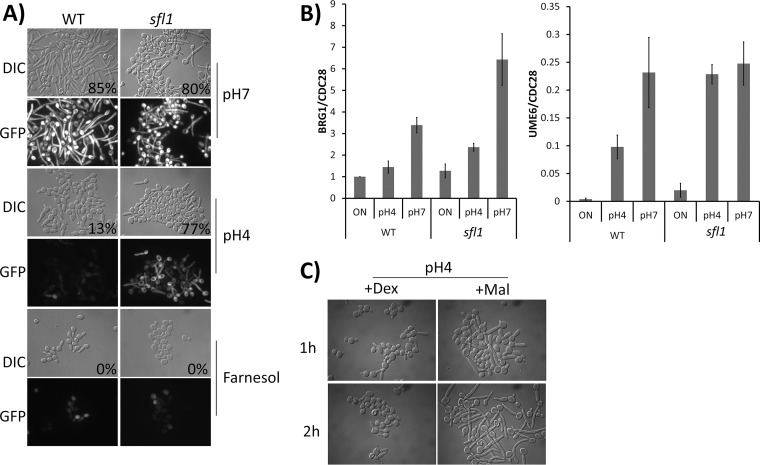
(A) Morphology of WT and *sfl1* strains expressing a copy of *HWP1p-*GFP after inoculation for 1 h in YPD medium set at pH 7 or pH 4 or supplemented with 100 μM farnesol. Percent filamentation is indicated at bottom right of DIC images. (B) qRT-PCR of *BRG1* and *UME6* transcripts after WT and *sfl1* cells were grown at pH 4 and pH 7 for 1 h. qPCR values were normalized to *CDC28* transcript levels for each sample. Presented data represent means ± SEM of results from 3 independent experiments. (C) Morphology of WT strain expressing a copy of *MAL2p-BRG1* after inoculation for up to 2 h in YEP medium at pH 4 with either dextrose (+Dex) or maltose (+Mal) as the carbon source.

To uncover mechanisms for acidic pH-mediated hyphal inhibition, we designed a screen to find mutants that can undergo hyphal initiation in acidic pH, but not in the presence of farnesol, and to identify only those which are not constitutively hyphal. We screened the collection of 165 transcription factor mutants (46). The *sfl1* mutant was the only one able to bypass acidic pH-mediated inhibition of hyphal initiation but not the inhibition by farnesol (Fig. 2A). *sfl1* cells were in yeast form before inoculation. Both the WT and *sfl1* mutant strains were transformed with the *HWP1p-GFP* reporter, and cells from an overnight culture were inoculated into pH 4 and pH 7 media at 37°C. Yeast-to-hypha transition was monitored by assay of both morphological changes and the appearance of green fluorescent protein (GFP). In the WT strain, *HWP1p-GFP* was expressed at pH 7 but not at pH 4. In the *sfl1* mutant, expression of *HWP1p-*GFP was observed at both pH 4 and pH 7 (Fig. 2A). In contrast to the results seen with acidic pH, farnesol inhibition of hyphal formation and *HWP1-GFP* expression was not bypassed by the *sfl1* mutant. In addition to inoculation, which removes farnesol inhibition, a temperature shift to 37°C is also required for the *sfl1* mutant to form hyphae in acidic pH. These observations, together with the results in Fig. 1, suggest that farnesol-mediated Nrg1 degradation, temperature-induced transcriptional repression of *NRG1*, and the acidic pH pathway are three independent forms of regulation that inhibit hyphal initiation. Deletion of *SFL1* specifically bypasses the acidic pH inhibition.

Sfl1 has been characterized as a hyphal repressor that binds to the promoters of key hyphal regulator genes *BRG1*, *SFL2*, *UME6*, and *TEC1* (38). Sfl1 was previously shown to repress filamentation through, at least, direct transcriptional repression of the *BRG1* and *SFL2* genes (38), and Brg1 and Sfl2 were found to activate hyphal development by inducing *UME6* expression (29, 38). To examine if Sfl1 is responsible for acidic pH-mediated inhibition of the expression of these hyphal regulators, we examined the transcriptional levels of key hyphal regulators *BRG1* and *UME6* in acidic and neutral pH. In the WT strain, the expression of *BRG1* and *UME6* was increased at pH 7, but not pH 4, during hyphal initiation (Fig. 2B). Deleting *SFL1* resulted in a higher increase of *BRG1* expression during hyphal initiation at pH 7, consistent with the reported repression of *BRG1* expression by Sfl1 (38). The increase in the level of *BRG1* expression in the *sfl1* mutant was found to be much lower at pH 4 than at pH 7, indicating additional Sfl1-independent repression of *BRG1* expression by acidic pH. Inactive Rim101 might be responsible for the observed repression at pH 4 (7). In comparison to *BRG1*, the increases in the levels of *UME6* expression were similar at pH 4 and pH 7 in the *sfl1* mutant. It is possible that *SFL1* deletion and temperature shift to 37°C function together to induce *SFL2* expression, which leads to *UME6* expression (38). Those data suggest that *BRG1* expression may be represent convergent point of regulation by multiple signaling pathways. Consistent with this, overexpressing *BRG1* restored hyphal initiation in acidic pH. Expressing *BRG1* under the control of the *MAL2* promoter, we observed the formation of hyphae when the cells were inoculated into maltose medium at pH 4 but not in glucose medium (Fig. 2C).

### The *hog1*, *mkc1*, and *cmk1* mutants also undergo hyphal initiation in acidic pH.

**FIG 3:**
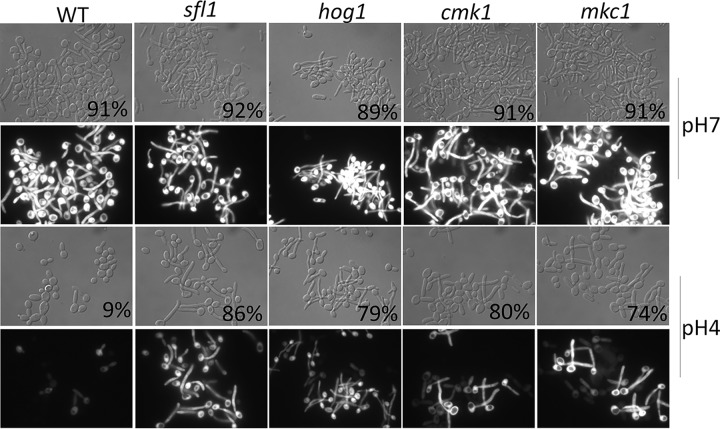
Morphology of WT, *sfl1*, *hog1*, *cmk1*, and *mkc1* strains expressing a copy of *HWP1p-*GFP after inoculation for 1 h in YPD medium set at pH 7 or pH 4. Filamentation percentages are indicated at bottom right of DIC images.

To identify potential regulators and pathways that regulate Sfl1, we applied the same screening conditions to the C. albicans kinase mutant collection containing 80 homozygous protein kinase and protein kinase-related gene deletion strains (47). From the screen, we identified the following four mutants which, similarly to *sfl1*, could filament at pH 4 and 37°C but not in the presence of farnesol: the core stress response genes *HOG1* and *PBS2* (39, 40), the cell wall integrity gene *MKC1* (43, 44), and the calcium/calmodulin-dependent kinase *CMK1* (48). Cmk1 is also involved in the regulation of cell wall integrity and oxidative stress response in C. albicans (49). All four mutants were in yeast form before inoculation and effectively developed germ tubes in acidic pH, but not in the presence of farnesol, as evidenced by morphology and the expression of *HWP1p-GFP* (Fig. 3). The *hog1* mutant was used as the representative for both *hog1* and *pbs2*, as they function in the same MAP kinase pathway and show similar phenotypes. This finding suggests a potential for functional interactions between these stress response kinases and Sfl1 in the regulation of hyphal development.

### Acidic pH sustains basal Hog1 phosphorylation after inoculation.

**FIG 4:**
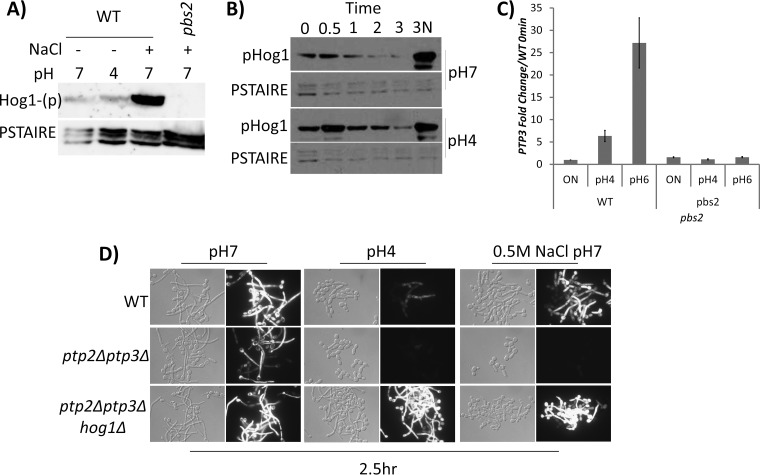
(A) Acidic pH does not induce Hog1 phosphorylation. A Phospho-Hog1 immunoblot of cells grown for 3 h to the logarithmic phase and inoculated into fresh YPD medium at pH 4, pH 7, or pH 7 with 0.5 M NaCl for 5 min is shown. A *pbs2* mutant strain was induced in 0.5 M NaCl as a negative control. A parallel blot was probed with anti-PSTAIRE as a loading control. (B) Acidic pH sustains Hog1 basal phosphorylation. A Phospho-Hog1 immunoblot of overnight cells (0 h) inoculated into fresh YPD medium at pH 4 and pH 7 for 3 h is shown. Aliquots were collected every hour. At 3 h, an aliquot of cells were shifted to medium with 1 M NaCl (3N) to induce Hog1 phosphorylation. A parallel blot was probed with anti-PSTAIRE as a loading control. (C) Acidic pH inhibits *PTP3* transcription. qRT-PCR of WT and *pbs2* cells was performed to measure the levels of *PTP3* transcript after cells were grown at pH 4 and pH 6 for 15 min. qPCR values were normalized to *ACT1* for each samples, and overnight (ON) samples were set to a value of 1. (D) Hog1 phosphorylation inhibits hyphal initiation. Morphology and *HWP1p*-GFP expression of WT, *ptp2 ptp3*, and *ptp2 ptp3 hog1* strains expressing a copy of *HWP1p-*GFP after inoculation for 2.5 h in YPD medium at pH 7 or pH 4 or supplemented with 0.5 M NaCl are shown.

Since loss of Hog1 phosphorylation rescued filamentation in acidic pH, we examined the effects of pH on Hog1 phosphorylation. Hog1 phosphorylation is regulated through either activation by the upstream MAP kinase (MAPK) kinase Pbs2 or dephosphorylation through the MAPK tyrosine phosphatases *PTP2* and *PTP3*. We excluded the first possibility, as inoculation of log-phase cells into yeast extract-peptone-dextrose (YPD) medium at pH 4 and pH 7 for 5 min did not induce Hog1 phosphorylation. In comparison, inoculating cells into 1 M NaCl for 5 min strongly induced Hog1 phosphorylation. As expected, Hog1 phosphorylation was absent in the *pbs2* mutant even in the presence of NaCl (Fig. 4A). We then examined if acidic pH could slow the dephosphorylation of Hog1. Hog1 basal phosphorylation increased during hyphal initiation but was found to have reduced to the initial basal level after 1 h at pH 7. In comparison, cells inoculated into acidic pH were slower to deplete basal Hog1 phosphorylation (Fig. 4B), This suggests that pH influences the duration of Hog1 phosphorylation. Since dephosphorylation is regulated by Hog1 phosphatases, we examined their transcript levels and observed pH-dependent expression of *PTP3*, with its transcript elevated in neutral pH and repressed in acidic pH (Fig. 4C).

### Hog1 phosphorylation inhibits hyphal initiation.

To demonstrate that the inhibitory effect of Hog1 on hyphal initiation happens via Hog1 phosphorylation, a *ptp2 ptp3* double mutant lacking both *PTP2* and *PTP3* phosphatases of Hog1 (32) was used to examine hyphal development at pH 7 or pH 4 in YPD medium or at pH 7 in YPD medium with 0.5 M NaCl (Fig. 4D). The *ptp2 ptp3* double mutant was able to develop hyphae at pH 7 but was completely defective in forming hyphae at pH 4 or with 0.5 M NaCl. Hyphal growth was not obviously impaired in the WT in 0.5 M NaCl but was completely blocked in the *ptp2 ptp3* double mutant under the same conditions. This suggests that Hog1 phosphorylation inhibits hyphal initiation. To further demonstrate that the function of Ptp2 and Ptp3 in hyphal initiation happens via Hog1 dephosphorylation, we examined a *ptp2 ptp3 hog1* triple mutant (32). Deletion of *HOG1* in the phosphatase double mutant completely alleviated the phenotype (Fig. 4D), affirming the role of Hog1 phosphorylation in the inhibition of hyphal initiation.

### NaCl retards hyphal initiation, and the effect is bypassed by the *sfl1* mutant.

**FIG 5:**
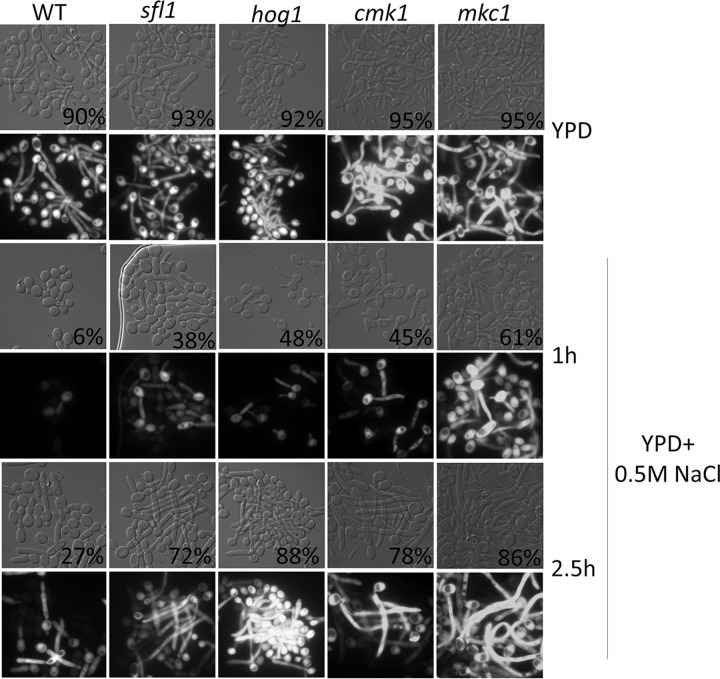
Morphology and GFP expression of WT, *sfl1*, *hog1*, *cmk1*, and *mkc1* strains expressing a copy of *HWP1p-*GFP inoculated for 1 h and 2.5 h in YPD medium supplemented with 0.5 M NaCl. Percent filamentation is indicated on bottom right of DIC images.

We next examined the functional relationships between Sfl1 and these kinases. If Sfl1 acts downstream of the kinases, we would expect that (i) conditions that activate the upstream kinases would be inhibitory to hyphal initiation and (ii) this inhibition could be reversed by loss of Sfl1 if it functions downstream of the kinase pathway. Hog1 is the best-studied kinase among the three, so we chose to activate Hog1 and evaluate the ability of the mutant strains to rescue this effect. Cells from overnight cultures were inoculated into media containing 0.5 M NaCl, and their ability to initiate hyphal growth was monitored. At 1 h, NaCl had a negative effect on hyphal initiation based on the low percentage of WT cells with initiated germ tube morphology and on the levels of *HWP1p-GFP* expression (Fig. 5). The inhibitory effect of NaCl was transient, as the WT strain had initiated and grown hyphae by 2.5 h. The *hog1* mutant had a significantly larger amount of germ tube morphology than the WT at 1 h. The improved filamentation was still present in the *hog1* mutants at 2.5 h.

Like the *hog1* mutant, the *sfl1*, *mkc1*, and *cmk1* mutants were able to bypass the negative effect of NaCl in hyphal initiation. They showed significantly higher percentages of germ tube morphology than the WT at 1 h, and all the mutants showed better hyphal growth at 2.5 h (Fig. 5). In particular, the *mkc1* mutant showed a stronger bypass of hyphal initiation in 0.5 M NaCl than the *sfl1* and *cmk1* mutants. Mkc1 is known to be activated under several stress conditions, and its phosphorylation is Hog1 dependent (43). Overall, the similar hyphal initiation phenotypes among the *sfl1* and three kinase mutants under conditions of acidic pH and 0.5 M NaCl indicate that the kinase may converge on Sfl1 to regulate the expression of hyphal transcription.

### Loss of Cmk1 and Sfl1 leads to tolerance of cation stress.

**FIG 6:**
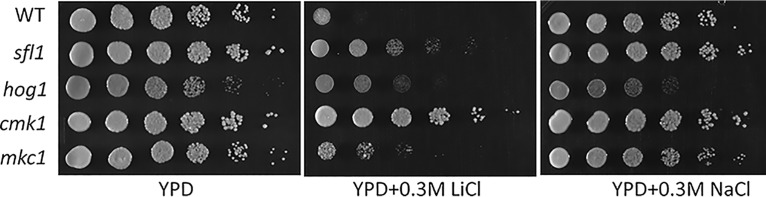
Cation stress sensitivity of the WT, *sfl1*, *hog1*, *cmk1*, and *mkc1* strains on YPD plates containing 0.3 M LiCl or 0.5 M NaCl.

There is limited information on the functions and regulation of Cmk1, and hyphal initiation represents the first evidence that places Sfl1 downstream of three stress-responsive kinases. In a phenotypic profiling of transcription factor mutants performed previously by Homann et al. (46), the *sfl1* mutant was found to have altered sensitivity to the cation stress of 0.3 M LiCl. To further evaluate whether Sfl1 would be found to be functionally linked to the stress-responsive kinases in a different setting, we examined growth sensitivity to LiCl. Growth levels of the WT and mutant strains were compared on YPD medium and on YPD medium plus 0.3 M LiCl or 0.5 M NaCl. The WT strain was sensitive to 0.3 M LiCl (Fig. 6). The *cmk1* mutant showed a strong tolerance of LiCl, and no growth differences were seen in the presence or absence of 0.3 M LiCl. The *sfl1* mutant also showed strong tolerance of LiCl compared to the WT and was able to grow in the presence of 0.3 M LiCl (Fig. 6). The other two mutant strains, *hog1* and *mkc1*, showed only a limited increase in LiCl tolerance in comparison to the WT. The increase in LiCl tolerance by the *hog1* mutant shown here was unexpected, as the mutant is highly sensitive to NaCl stress (Fig. 6) (50). The growth tolerance of the *cmk1* and *sfl1* mutants, as well as of the *hog1* mutant to a certain extent, in the presence of LiCl provides a functional assay different from hyphal initiation. This suggests close relationships in function and regulation among Cmk1, Hog1, and Sfl1.

## DISCUSSION

**FIG 7:**
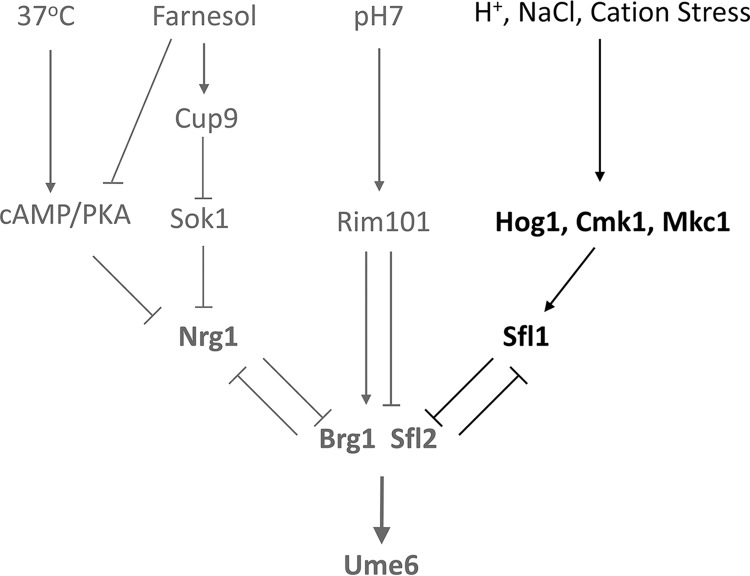
Model of signal integration during hyphal initiation.

The process of hyphal development is important for survival of and infection by C. albicans in the host. On the other hand, host microenvironments regulate hyphal development (51). While it has long been known that acidic pH suppresses hyphal development, the details of how it affects hyphal initiation are still elusive. In this study, we showed that Sfl1 transcriptional repression and acidic pH inhibit hyphal initiation by retarding the expression of *BRG1* and *UME6*, key regulators of hyphal development. Sfl1 and acidic pH act independently of the published farnesol- and cAMP/PKA-regulated Nrg1 downregulation pathways (Fig. 7). Nrg1 is downregulated and dissociated from the *HWP1* promoter at both pH 4 and pH 7. The *sfl1* mutant completely bypasses acidic pH but is still sensitive to farnesol inhibition and is unable to initiate hyphae without a temperature shift to 37°C. We noticed that *BRG1* expression was not fully induced in the *sfl1* mutant at pH 4 in comparison to pH 7, indicating Sfl1-independent regulation of *BRG1* by pH. Since the pH-responsive Rim101 transcription factor is the major regulator of pH-responsive genes and contributes to the repression of hyphal development at acidic pH (7, 14, 52), the inactive Rim101 could be responsible for the observed repression of *BRG1* at pH 4 (Fig. 7). Unlike *BRG1* expression, *UME6* expression was induced similarly at pH 4 and pH 7 in the *sfl1* mutant. It is possible that *SFL1* deletion and the temperature shift to 37°C functioned together to increase *SFL2* expression, leading to *UME6* expression (Fig. 7) (38). We place Sfl2 and Brg1 together in our model of hyphal initiation because expression of both required a temperature shift to 37°C. Likewise, deletion of either *SFL2* or *BRG1* in the *sfl1* mutant similarly impaired hyphal formation (38). We suggest that *BRG1* expression may be a convergent point of regulation by Sfl1 and Rim101 under acidic pH conditions. This is consistent with a previous report by Su et al. indicating that *N*-acetylglucosamine, serum, or neutral pH can promote hyphal development in log-phase cells, without inoculation, by activating the expression of Brg1 (53). Also, Brg1 represses *NRG1* expression and gradually reduces Nrg1 levels after a few hours of growth, as Nrg1 and Brg1 are negative regulators of each other (33). Unlike *nrg1* cells, which are constitutively hyphae, *sfl1* cells are in yeast form under many yeast growth conditions. The ability of the *sfl1* deletion mutant to bypass acidic pH is specific as it is the only mutant from the transcription factor deletion collection to have been found to bypass acidic pH in our screen. Several genes that are upregulated under the *sfl1* deletion conditions have promoters which are bound by Sfl1/Sfl2 (38) and are repressed by Rim101 (14). We suggest that Rim101 and Sfl1/Sfl2 may function together. Robust hyphal initiation requires downregulation of both the Nrg1 and Sfl1 transcriptional repressors (Fig. 7).

This study showed that acidic pH and NaCl inhibit or retard hyphal initiation via the activity of stress-responsive kinases and Sfl1 (Fig. 7). It is important to point out the temporal differences between the two signals that increase Hog1 phosphorylation and their effects on hyphal initiation. NaCl induced a strong and fast Hog1 phosphorylation response, leading to only a delay in hyphal initiation. Inoculating cells into fresh media induced a rise in basal Hog1 phosphorylation, and this increase in basal Hog1 phosphorylation was prolonged at pH 4, leading to inhibition of hyphal initiation. In a previous study, we found that 3 to 5 h after hyphal initiation in rich medium, basal Hog1 phosphorylation repressed the expression of *BRG1* via the transcriptional repressor Sko1 and hyphal cells were converted back to yeast (32). The *sko1* mutant can sustain hyphae in rich medium but cannot bypass the acidic pH inhibition of hyphal initiation. The *hog1 brg1* mutant behaved like *brg1* (32), placing Brg1 downstream of Hog1. The three timings of Hog1 phosphorylation have very different effects on initiation. NaCl induces a strong Hog1 phosphorylation, but the effect is short in duration. NaCl inhibited hyphal development in the *ptp2 ptp3* mutant only when Hog1 phosphorylation was sustained. The effect of Hog1 phosphorylation timing on hyphal initiation supports our model for a “window of opportunity” (28, 29). Hyphal development needs a sufficient level of Brg1 during the time window when Nrg1 dissociates from the promoters of hypha-specific genes. We also showed that transient activation/phosphorylation of Hog1 in response to NaCl retards hyphal initiation and that this effect can be alleviated by *Sfl1* and all three kinase mutants. Although we do not have direct evidence indicating that Hog1 regulates Sfl1 in C. albicans, studies in Saccharomyces cerevisiae have shown a functional link between Sfl1 and Hog1. Hog1 and Sfl1 are required for expression of the aquaporin gene *AQY2* (54). S. cerevisiae Sfl1 (*Sc*Sfl1) is phosphorylated at Ser556 by Hog1 in response to stress (55), and the phosphor residue (Thr602) is conserved in C. albicans Sfl1 (*Ca*Sfl1). Interestingly, Hog1 is activated in response to citric acid and is important for adaption to citric acid stress (56). Cmk1 is also involved in a weak acid response, as loss of Cmk1 results in weak organic acid resistance (57). Consistent with the studies in S. cerevisiae, our data also suggest the involvement of Cmk1, Hog1, and Sfl1 in response to cation stress by C. albicans. Acidic pH, weak acids, cation stress, and osmotic stresses are likely encountered by C. albicans in hosts. Therefore, these stress-responsive kinases and Sfl1 are important in controlling hyphal development and virulence *in vivo* (36).

## MATERIALS AND METHODS

### Plasmid and strain construction.

The C. albicans strains used in this study are listed in Table S1 in the supplemental material and primers in Table S2. To generate *HWP1p*-*GFP-SAT1* (plasmid 1254), primers HWP1p-GFP-NAT F/R was used to PCR amplify the genomic sequence 1 kb upstream of the *HWP1* transcription start site. The fragment was incorporated into the plasmid by the use of Gibson assembly (58). The resulting plasmid was linearized by digestion with AflII and was integrated into the endogenous *HWP1* promoter in the different strains, and successful transformants were selected on YPD medium plus 200 μg/ml nourseothricin.

10.1128/mSphere.00672-19.1 TABLE S1 The C. albicans strains used in this study. Download Table S1, PDF file, 0.09 MB. Copyright © 2020 Unoje et al. 2020 Unoje et al. This content is distributed under the terms of the Creative Commons Attribution 4.0 International license.

10.1128/mSphere.00672-19.2 TABLE S2 Primers used in this study. Download Table S2, PDF file, 0.04 MB. Copyright © 2020 Unoje et al. 2020 Unoje et al. This content is distributed under the terms of the Creative Commons Attribution 4.0 International license.

### Media and growth conditions.

C. albicans strains were grown in yeast extract-peptone (2% Bacto peptone, 1% yeast extract, 0.015% l-tryptophan) with 2% dextrose or maltose (for promoter shutdown assay) as a carbon source at 30°C to saturation (optical density at 600 nm [OD_600_] = 10 to 12, ∼17 h). To induce hyphae, the saturated cultures were inoculated 1:50 into YPD media prewarmed to 37°C and supplemented with the necessary stressor (HCl to pH 4, NaCl, farnesol). Aliquots of growing cultures were collected at the indicated time point and washed once with water before viewing differential inference contrast (DIC) and fluorescein isothiocyanate (FITC) fluorescence with a microscope was performed. Percentages of filamentation were determined by counting a total of 300 cells per experiment, and each experiment was conducted 3 times.

### Mutant collection screening.

To identify mutants that filament in acidic pH, the kinase mutant collection generated by Blankenship et al. (47) and the transcription regulator mutant collection generated by Homann et al. (46) were used for screening. Mutants were grown in 96-well plates overnight in YPD medium until saturation and inoculated 1:50 into 100 μl fresh YPD medium (pH 4) prewarmed to 37°C for 90 min and viewed under a microscope to observe filamentation. Filamentous strains were confirmed first in 1 ml in 24-well plates and then in 10 ml in glass flasks shaken at 200 rpm in a 37°C water bath. Mutants that were filamentous in overnight culture were excluded from further analysis.

### Stress sensitivity assay.

Strains were grown at 30°C to saturation and a 5-fold serial dilution, starting with 10^3^ cells, was spotted onto YPD medium with or without 0.3 M LiCl. The plates were grown at 30°C for 36 h before imaging.

### Lysate extraction and immunoblotting.

For phospho-Hog1 detection, cells were grown to saturation and inoculated 1:50 into fresh medium at pH 7 or pH 4. At each time point, aliquots were collected in a 50-ml conical tube with ice and centrifuged for 3 min at 4°C, and the cell pellets were flash frozen in liquid nitrogen and stored until cell lysis. The pellets were lysed by resuspension in kinase buffer (50 mM Tris-HCl [pH 7.5], 150 mM NaCl, 10% glycerol, 1% Triton X-100, 0.1% SDS, 5 mM EDTA, 50 mM EGTA, 50 mM sodium fluoride, 0.1 mM sodium orthovanadate, 10 mM sodium pyrophosphate, 1 mM phenylmethylsulfonyl fluoride [PMSF]) with glass beads and vigorously smashed using a Fast-Prep system (FP120; Thermo Electron, Waltham, MA) for four 20-s intervals with cooling on ice for 5 min between the intervals. The crude lysate was centrifuged to separate the lysate from debris, normalized, resolved by SDS-PAGE on an 8% gel, and transferred to a nitrocellulose membrane. Phospho-Hog1 levels were determined by blocking with PBST (phosphate-buffered saline with Tween 20)–5% BSA (bovine serum albumin) for 1 h and probing with anti-phospho-p38 antibody in PBST plus BSA overnight at 4°C. The membranes were washed and then probed with an anti-rabbit IgG secondary antibody in PBST–3% milk for 1 h. For determination of total Hog1 levels, the membranes were blocked for 1 h in PBST–3% milk for 1 h and probed with an anti-Myc primary antibody that had been preconjugated with horseradish peroxidase (HRP) for 1 h. A control blot for PSTAIRE was done using a rabbit polyclonal primary antibody (Roche) and a goat anti-rabbit HRP-conjugated secondary antibody (Bio-Rad).

### Promoter shutdown assay.

To observe the rate of degradation of Nrg1-Myc, CAI4 cells containing a copy of *MAL2p-NRG1-13xMYC* were grown overnight in yeast extract-peptone (YEP) medium plus 2% maltose to overexpress Nrg1-Myc and then inoculated at 1:50 into fresh YEP medium plus 2% dextrose for 1 h to shut down the activity of the *MAL2* promoter. Aliquots were collected at each time point and centrifuged at 3,500 rpm, the supernatant was aspirated, and the cell pellets were flash frozen in liquid nitrogen until cell lysis. The pellets were lysed by resuspension in lysis buffer and were vigorously smashed with glass beads in a Fast-Prep system (FP120; Thermo Electron, Waltham, MA) for four 20-s intervals with cooling on ice for 5 min between intervals. The crude lysate was centrifuged to separate the lysate from debris, normalized, resolved by SDS-PAGE on an 8% gel, and transferred to a nitrocellulose membrane. Nrg1 levels were determined by probing the membrane with an HRP-conjugated mouse monoclonal antibody (Roche) against the c-Myc epitope. A control blot for PSTAIRE was done using a rabbit polyclonal primary antibody (Roche) and a goat anti-rabbit HRP-conjugated secondary antibody (Bio-Rad).

### Quantitative RT-PCR.

RNA was extracted from yeast and hyphal cells using a Zymo Quick-RNA MiniPrep kit, and 2 μg was reverse transcribed into cDNA using a Bio-Rad iScript reverse transcription (RT) kit. Quantitative PCR was performed on a Bio-Rad iCycler using Bio-Rad SYBR green reaction mix and the corresponding primers. The cycle parameters were 95°C for 1 min and 39 cycles of 95°C for 10 s, 56°C for 45 s, and 68°C for 20 s.
