# Supplementary material for: Linking Sfl1 Regulation of Hyphal Development to Stress Response Kinases in Candida albicans
Source: mSphere. 2020 Jan 15;5(1):e00672-19. doi: 10.1128/mSphere.00672-19 (PMC6968649; doi:10.1128/mSphere.00672-19)
Supplement: TABLE S1 [file mSphere.00672-19-st001.pdf]

Strain	Genotype	Source
CAI4	*ura3Δ::imm^434/ura3Δ::imm^434*	1
hog1	*ura3Δ::imm^434/ura3Δ::imm^434, his1Δ::hisG/his1Δ::hisG, arg4Δ::hisG /arg4Δ::hisG, hog1Δ::ARG4/ hog1Δ::URA3*	2
pbs2	*ura3Δ::imm^434/ura3Δ::imm^434, his1Δ::hisG/his1Δ::hisG, arg4Δ::hisG /arg4Δ::hisG, pbs2Δ::ARG4/ pbs2Δ::URA3*	2
sfl1	*his1Δ/his1Δ, leu2Δ/ leu2Δ, arg4Δ/arg4Δ, ura3Δ/ura3Δ::imm^434, iro1/iro1Δ::imm^434, sfl1Δ::HIS1/sfl1Δ::LEU2*	3
rim101	*his1Δ/his1Δ, leu2Δ/ leu2Δ, arg4Δ/arg4Δ, ura3Δ/ura3Δ::imm434, iro1/iro1Δ::imm434, rim101Δ::HIS1/rim101Δ::LEU2*	3
ptp2ptp3	*his1Δ/his1Δ, leu2Δ/ leu2Δ, arg4Δ/arg4Δ, ura3Δ/ura3Δ::imm^434, iro1/iro1Δ::imm^434, ptp3Δ::C.dubliniensis HIS1/ptp3Δ::C.maltosa LEU2, ptp2Δ::URA3/ptp2Δ::ARG41*	4
ptp2ptp3hog1	*his1Δ/his1Δ, leu2Δ/ leu2Δ, arg4Δ/arg4Δ, ura3Δ/ura3Δ::imm^434, iro1/iro1Δ::imm434, ptp3Δ::C.dubliniensis HIS1/ptp3Δ::C.maltosa LEU2, ptp2Δ::ura3Δ/ptp2Δ::ARG4, hog1Δ::URA3/ hog1Δ::FRT*	4
CAI4 + HWP1p-GFP	*ura3Δ::1 imm^434/ura3Δ:1 imm^434, HWP1p/HWP1p-GFP-SAT1*	this study
hog1 + HWP1p-GFP	*ura3Δ::imm^434/ura3Δ::imm^434, his1Δ::hisG/his1Δ::hisG, arg4Δ::hisG /arg4Δ::hisG, hog1Δ::ARG4/ hog1Δ::URA3, HWP1p/HWP1p-GFP-SAT1*	this study
pbs2 + HWP1p-GFP	*ura3Δ::imm^434/ura3Δ::imm^434, his1Δ::hisG/his1Δ::hisG, arg4Δ::hisG /arg4Δ::hisG, pbs2Δ::ARG4/ pbs2Δ::URA3, HWP1p/HWP1p-GFP-SAT1*	this study
sfl1 + HWP1p-GFP	*his1Δ/his1Δ, leu2Δ/ leu2Δ, arg4Δ/arg4Δ, ura3Δ/ura3Δ::imm434, iro1/iro1Δ::imm434, sfl1Δ::HIS1/sfl1Δ::LEU2, HWP1p/HWP1p-GFP-SAT1*	this study
ptp2ptp3 + HWP1p-GFP	*his1Δ/his1Δ, leu2Δ/ leu2Δ, arg4Δ/arg4Δ, ura3Δ/ura3Δ::imm434, iro1/iro1Δ::imm434, ptp3Δ::C.dubliniensis HIS1/ptp3Δ::C.maltosa LEU2, ptp2Δ::URA3/ptp2Δ::ARG4, HWP1p/HWP1p-GFP-SAT1*	this study
ptp2ptp3hog+ HWP1p-GFP	*his1Δ/his1Δ, leu2Δ/ leu2Δ, arg4Δ/arg4Δ, ura3Δ/ura3Δ::imm434, iro1/iro1Δ::imm434, ptp3Δ::C.dubliniensis HIS1/ptp3Δ::C.maltosa LEU2, ptp2Δ::ura3Δ/ptp2Δ::ARG4, hog1Δ::URA3/ hog1Δ::FRT, HWP1p/HWP1p-GFP-SAT1*	this study
CAI4+MAL2 p-NRG1-MYC	*ura3Δ::imm^434/ura3Δ::imm^434, ADE2/ade2Δ::MAL2p-NRG1-13MYC-URA3*	4

	**References**
1	Fonzi WA, Irwin MY. 1993. Isogenic strain construction and gene mapping in *Candida albicans*. Genetics **134**:717–28.
2	Blankenship JR, Fanning S, Hamaker JJ, Mitchell AP. 2010. An extensive circuitry for cell wall regulation in *Candida albicans*. PLoS Pathog **6**:e1000752.
3	Homann OR, Dea J, Noble SM, Johnson AD. 2009. A phenotypic profile of the *Candida albicans* regulatory network. PLoS Genet **5**:e1000783.
4	Su C, Lu Y, Liu H. 2013. Reduced TOR signaling sustains hyphal development in *Candida albicans* by lowering Hog1 basal activity. Mol Biol Cell **24**:385–97.
5	Lu Y, Su C, Wang A, Liu H. 2011. Hyphal development in *Candida albicans* requires two temporally linked changes in promoter chromatin for initiation and maintenance. PLoS Biol **9**:e1001105.
