# Supplementary material for: Linking Sfl1 Regulation of Hyphal Development to Stress Response Kinases in Candida albicans
Source: mSphere. 2020 Jan 15;5(1):e00672-19. doi: 10.1128/mSphere.00672-19 (PMC6968649; doi:10.1128/mSphere.00672-19)
Supplement: TABLE S2 [file mSphere.00672-19-st002.pdf]

Supplemental Table 2

Primers used for qRT-PCR

ACT1 qPCR(F): TGGTGATGGTGTTACTCACG

ACT1 qPCR (R): GACAATTTCTCTTTCAGCAC

CDC28 qPCR (F): TGGATTAGCTCGAGCATTTG

CDC28 qPCR (R): GGGTAGATATGTGGTCTGTTGG

NRG1 qPCR (F): GAATTCAAACCATCAACCAA

NRG1 qPCR (R): TGATTGTTGTGACAATGGAG

BRG1 qPCR (F): AGCTGGTGTGCCACCTCCAC

BRG1 qPCR (R): TACCACACCTGTGACATCTG

UME6 qPCR (F): TCTTACCTCAATCAGCATTA

UME6 qPCR (R):CAGCACTAACACTGACACC

Primers used for HWP1p-GFP Cloning:

HWP1p-GFP-NAT (F):

GCCAGTGAATTGTAATACGACTCACTATAGGGCGAATTGCCTTACACGCACA

TAAATTGC

HWP1p-GFP-NAT R:

ACACCAGTGAATAATTCTTCACCTTTAGACATTTTAATAATTGACGAAACTAA

AAGC
